# Signal amplification by reversible exchange for COVID-19 antiviral drug candidates

**DOI:** 10.1038/s41598-020-71282-6

**Published:** 2020-08-31

**Authors:** Hye Jin Jeong, Sein Min, Heelim Chae, Sarah Kim, Gunwoo Lee, Sung Keon Namgoong, Keunhong Jeong

**Affiliations:** 1 Department of Chemistry, Korea Military Academy, Seoul, 01805 South Korea; 2 Department of Chemistry, Seoul Women’s University, Seoul, 01797 South Korea

**Keywords:** Analytical chemistry, Medical and clinical diagnostics, NMR spectroscopy

## Abstract

Several drug candidates have been proposed and tested as the latest clinical treatment for coronavirus pneumonia (COVID-19). Chloroquine, hydroxychloroquine, ritonavir/lopinavir, and favipiravir are under trials for the treatment of this disease. The hyperpolarization technique has the ability to further provide a better understanding of the roles of these drugs at the molecular scale and in different applications in the field of nuclear magnetic resonance/magnetic resonance imaging. This technique may provide new opportunities in diagnosis and research of COVID-19. Signal amplification by reversible exchange-based hyperpolarization studies on large-sized drug candidates were carried out. We observed hyperpolarized proton signals from whole structures, due to the unprecedented long-distance polarization transfer by *para*-hydrogen. We also found that the optimal magnetic field for the maximum polarization transfer yield was dependent on the molecular structure. We can expect further research on the hyperpolarization of other important large molecules, isotope labeling, as well as polarization transfer on nuclei with a long spin relaxation time. A clinical perspective of these features on drug molecules can broaden the application of hyperpolarization techniques for therapeutic studies.

## Introduction

Coronavirus pneumonia (COVID-19) is a serious respiratory infectious disease that has emerged recently. Patients with coronavirus infection demonstrate fever with body temperature exceeding 38 °C, and symptoms, such as dry cough, fatigue, dyspnea, difficulty in breathing, and frosted glass-like symptoms in the lungs^1^. The disease is known to be highly transmittable without the occurrence of severe symptoms. The number of cases has reached over tens of millions worldwide.

To overcome this pandemic, many researchers have started working on developing a vaccine. However, ongoing vaccine developments are estimated to take over a year before becoming available for public use. As a result, the global clinical community is attempting to repurpose existing drugs to tackle the COVID-19 crisis. Recently, several drugs that can inhibit specific functions of the virus have been proposed and tested as part of the latest clinical treatment approaches. These drugs include chloroquine and hydroxychloroquine. Although the role of these drugs in the treatment of COVID-19 is controversial, hydroxychloroquine is more soluble than chloroquine and produces relatively less toxic metabolites^2–4^. Although chloroquine (7-chloro-4-(4-diethylamino-1-methylbutylamino)-quinoline) and hydroxychloroquine (2-[4-[(7-chloroquinolin-4-yl)amino]pentyl-ethylamino]ethanol), which have been in clinical usage for the last seventy years, are drugs for the treatment of autoimmune disease, they are also used as antimalarial drugs. These compounds have recently been reported as potential broad-spectrum antiviral drugs for COVID-19^5^; however, their benefits against COVID-19 are controversial, with no evident effect on hospitalized patients^6^. From a molecular perspective, these drugs are reported to inhibit viral infection by increasing the endosomal pH required for virus and cell fusion and interfere with the glycosylation of the cellular receptors of SARS-CoV^7^. They also play a role in the immune-modulating activity, potentially having a synergistic antiviral effect in vivo^8^. Chloroquine is administered orally, upon which it is distributed across the whole body, including the lungs. The EC_90_ value of chloroquine against 2019-nCoV in Vero E6 cells was 6.90 μM. This could be clinically achieved after a 500 mg dosage^9,10^. For COVID-19 treatment, further in vivo studies investigating dosage and/or a dynamic distribution under specific clinical conditions may be warranted.

Ritonavir/lopinavir (1,3-thiazol-5-ylmethyl *N*-[(2S,3S,5S)-3-hydroxy-5-[[(2S)-3-methyl-2-[[methyl-[(2-propan-2-yl-1,3-thiazol-4-yl)methyl]carbamoyl]amino]butanoyl]amino]-1,6-diphenylhexan-2-yl]carbamate/(2S)-N-[(2S,4S,5S)-5-[[2-(2,6-dimethylphenoxy)acetyl]amino] -4-hydroxy-1,6-diphenylhexan-2-yl]-3-methyl-2-(2-oxo-1,3-diazinan-1-yl)butanamide), the combination drug marketed as Kaletra, is a relatively new medication for the treatment and prevention of HIV. This compound has also been suggested as a potential drug candidate against COVID-19 due to its role as a proteinase inhibitor in association with the polyprotein processing of the coronavirus^11,12^. Recent reports have provided evidence in favor of this drug for the treatment of COVID-19, however, its beneficial effects remain a topic of controversy^13^. It is known to lessen viral loads and improve clinical symptoms^14,15^. However, compared to chloroquine and hydroxychloroquine, the interactions of this drug in the body at the molecular scale have not been elucidated so far.

Favipiravir (5-fluoro-2-oxo-1H-pyrazine-3-carboxamide), which has been approved in Japan for the treatment of influenza since 2014^16,17^, has also shown effectiveness in accelerating viral clearance in Chinese trials of hundreds of patients^18^.

To further understand the interactions of drugs with proteins, their metabolism, and other activities, nuclear magnetic resonance (NMR) spectroscopy has been widely used in pharmacokinetics. However, NMR is an inherently insensitive technique due to the small population differences in the spin states. Hyperpolarization, which generates a non-Boltzmann distribution of the spin state populations, may provide a breakthrough in addressing this challenge. Moreover, magnetic resonance imaging (MRI) is used as a solution to understand drug distribution throughout the body in vivo and study its activity. To visualize MRI signals, the drug must be tagged with specific compounds, such as chelating agents (e.g., Gd-chelate and Mn-chelate) with T1/T2 contrast. However, the fusion of additional compounds with the drug may have unpredictable effects due to their different molecular structures. This limitation may be overcome by using the hyperpolarization technique, which enhances the visualization of the hyperpolarized signal through MRI. The use of this state-of-the-art technology can address several challenges and could be a key to visualizing and understanding the in vivo real-time distribution and activity of drugs. This may provide an opportunity to further elucidate the antiviral activities of drugs used in the COVID-19 therapy.

Of the several methods to hyperpolarize drugs, the *para*-hydrogen-based signal amplification by reversible exchange (SABRE) method is the most promising for the hyperpolarization of several key structures with nitrogen. Although a remarkably high signal amplification is achieved using dynamic nuclear polarization, it requires extreme conditions (low temperature and high magnetic field) and a long hyperpolarization time (more than 2–3 h). *Para*-hydrogen-induced polarization provides a much higher signal enhancement than SABRE; however, molecules cannot be hyperpolarized continuously by parahydrogen. In this context, SABRE does not require harsh conditions, and substrates can be constantly hyperpolarized without any structural changes during the polarization transfer. Furthermore, in this method, the polarization can be real-time transferred from protons to other isotopes such as ^13^C^19^, ^15^N^20–23^, ^31^P^24^, ^19^F^25^, ^119^Sn^26^, and ^29^Si^27^ without chemical changes.

Recently, several breakthrough studies that develop hyperpolarized drugs or metabolites^28–32^, including those in pharmacokinetics, confirmed that SABRE^33^ could be useful for a wide-scale application. This is particularly important at a time when scientific remedies are the only means to conquer this pandemic. However, hyperpolarized drugs or metabolites are limited to small-sized molecules, which constrain their practical applications in many studies. Hyperpolarization studies using SABRE may be useful and important in facilitating future applications of MRI scanning using hyperpolarized COVID-19 drug candidates, which mostly have high molecular weights. It is anticipated that SABRE-based hyperpolarization studies on such large molecules may enable further research on a higher number of drugs and metabolites. To the best of our knowledge, this study is the first to evaluate the SABRE-based hyperpolarization of specific COVID-19 drug candidates in real-time.

## Results and discussion

### Favipiravir SABRE

**Figure 1:**
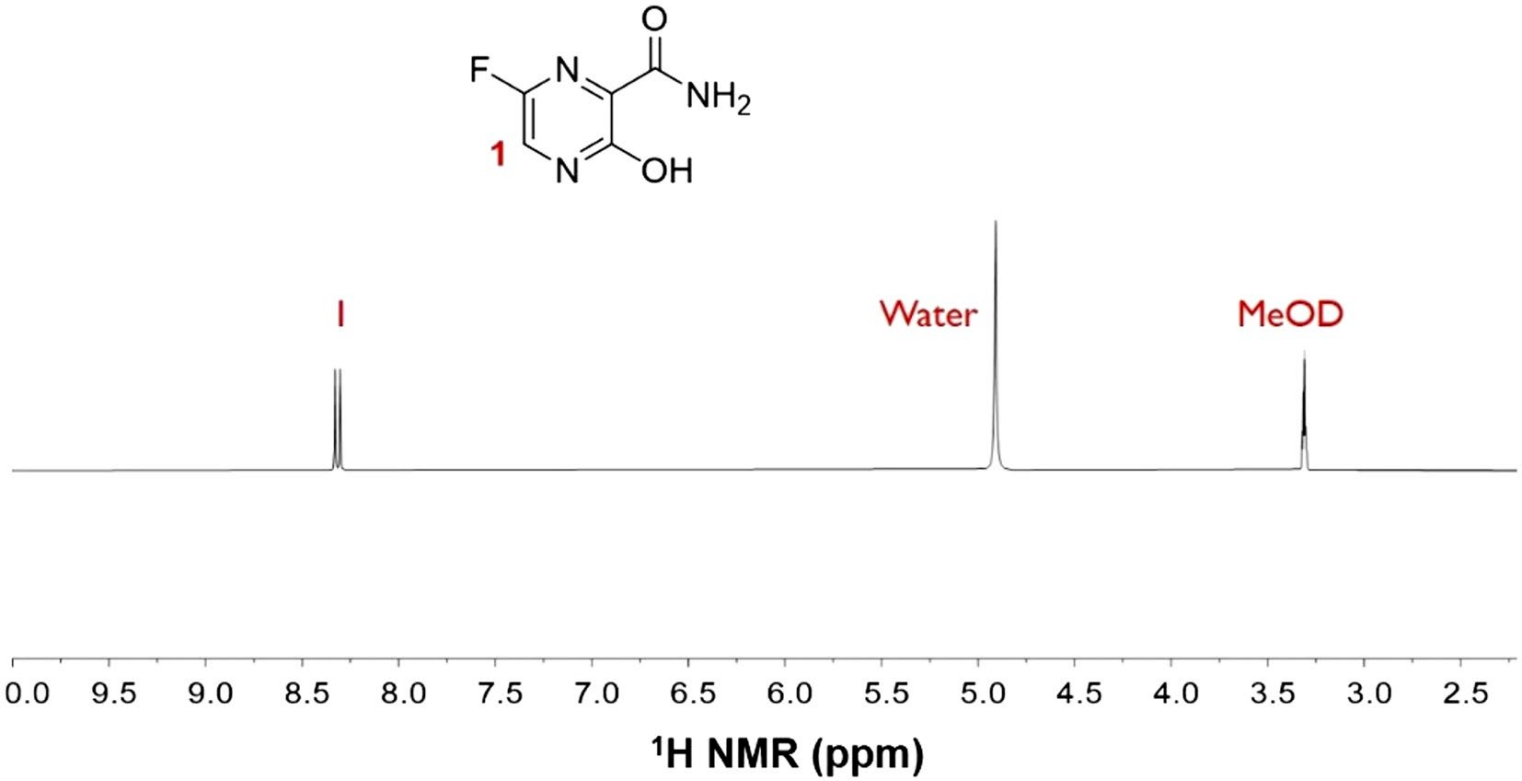
Favipiravir molecular structure and the normal ^1^H NMR signal (30° pulse, single scan in 300 MHz NMR).

The pyrazine moiety in favipiravir is recognized as the polarization source for SABRE^34^. However, its complex structure with several functional groups (fluorine, alcohol, and amide) has not been previously examined for SABRE hyperpolarization (Fig. 1).

**Figure 2:**
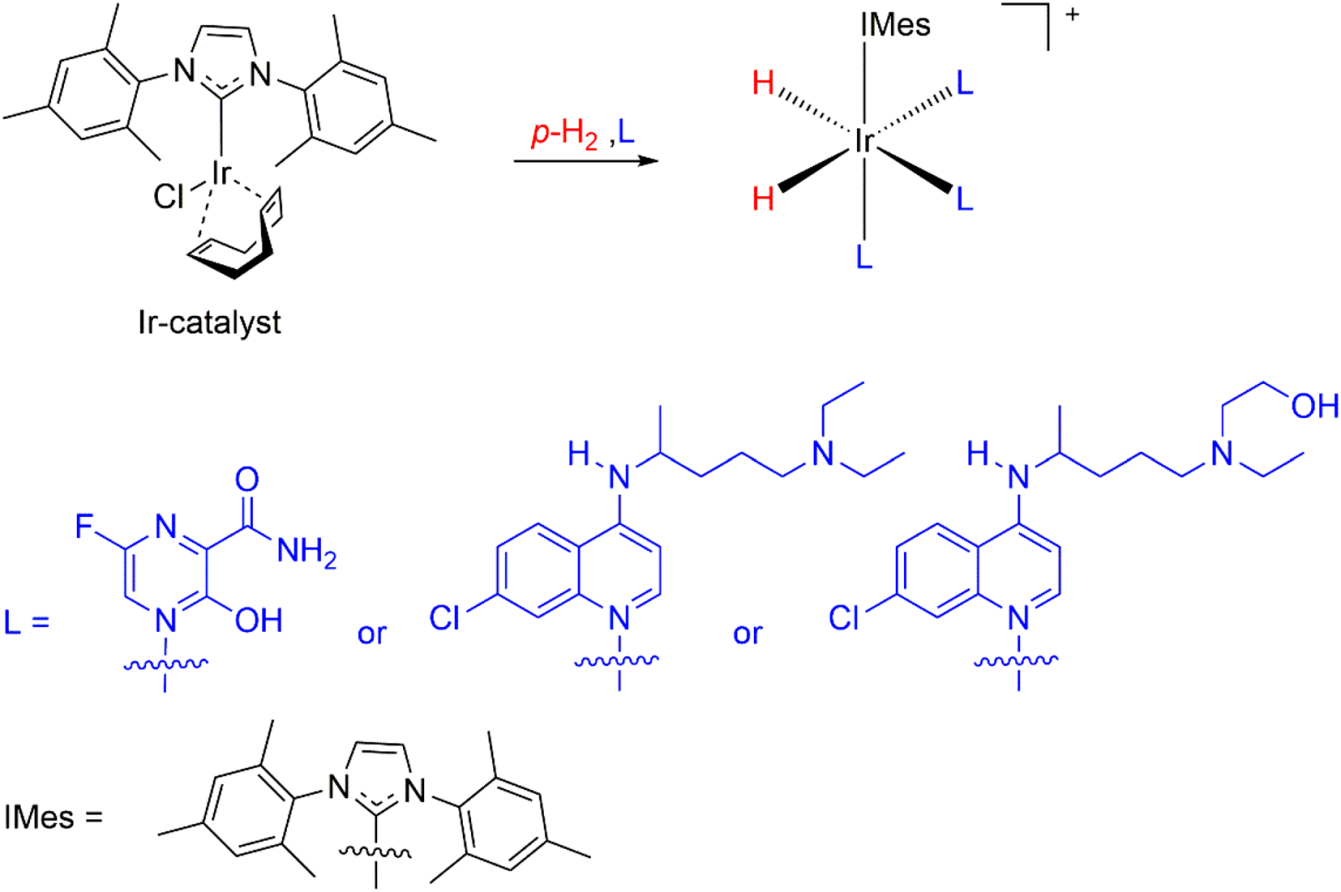
Ir catalyst and Chloroquine (hydroxychloroquine) / Favipiravir complex structures for SABRE in methanol. (wiggle denotes binding with the Ir-catalyst and L represents the binding drug structures).

**Figure 3:**
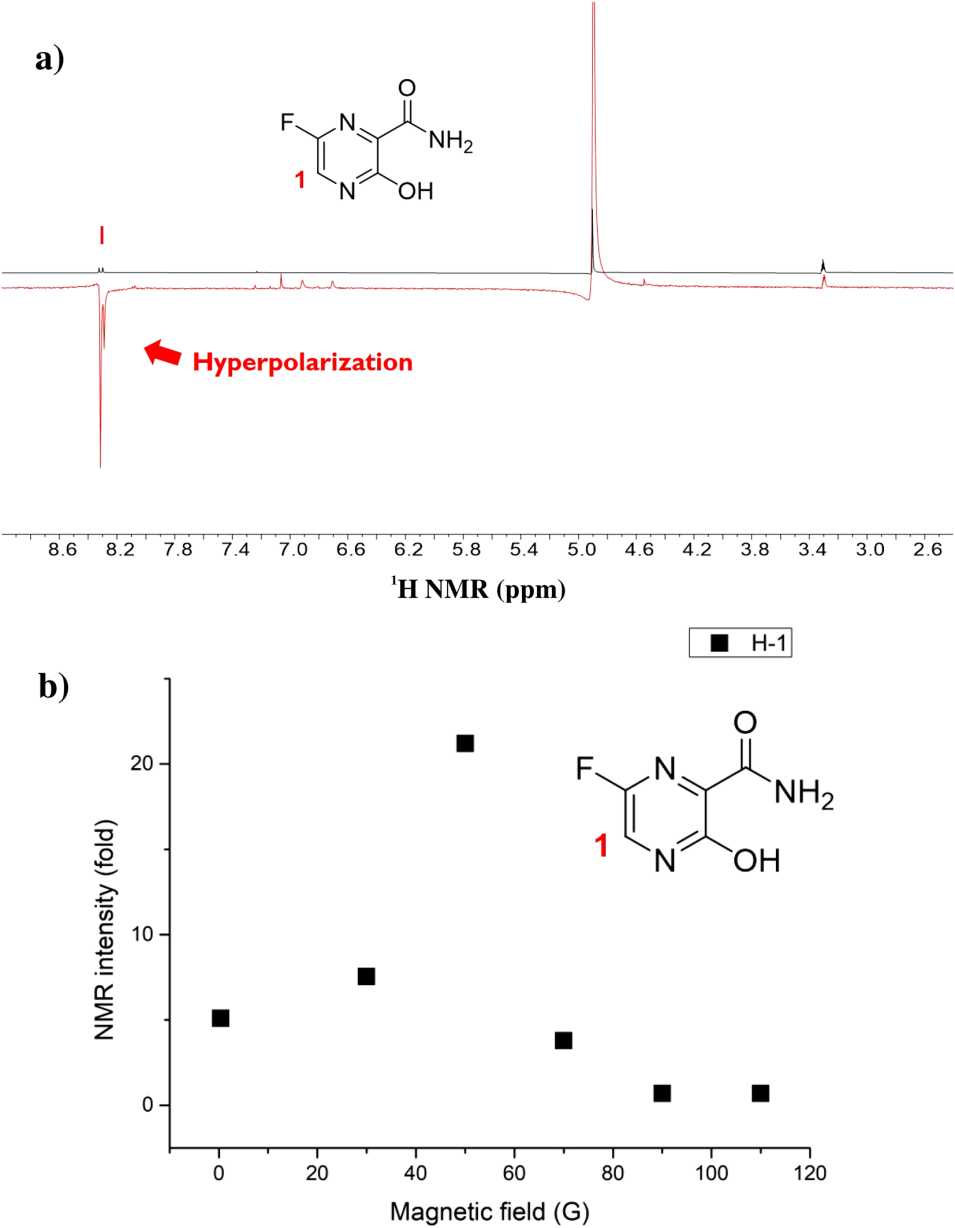
(**a**) Hyperpolarized signals from favipiravir after SABRE in the presence of a 50 G magnetic field; and (**b**) Amplification number of H-1 from the hyperpolarized favipiravir structure (single scan in 300 MHz ^1^H NMR, 30° pulse of ^1^H after polarization in each magnetic field).

Figure 2 depicts an estimate of its complex with an Ir catalyst, forming an exchangeable bond between Ir(I) and the pyrazine moiety. This form is expected for favipiravir to transfer polarization from *para*-hydrogen. As predicted, we successfully obtained a hyperpolarized signal from the aromatic proton of favipiravir with a ~ 20-fold enhanced signal after SABRE. An additional polarized signal was observed around 6–7 ppm and was attributed to the protons in the hydroxy and amide groups of the structure. The SABRE-based polarization trend with the magnetic field was maximized at approximately 50 G, which is consistent with previous reports (Fig. 3)^35–37^.

### Chloroquine SABRE

**Figure 4:**
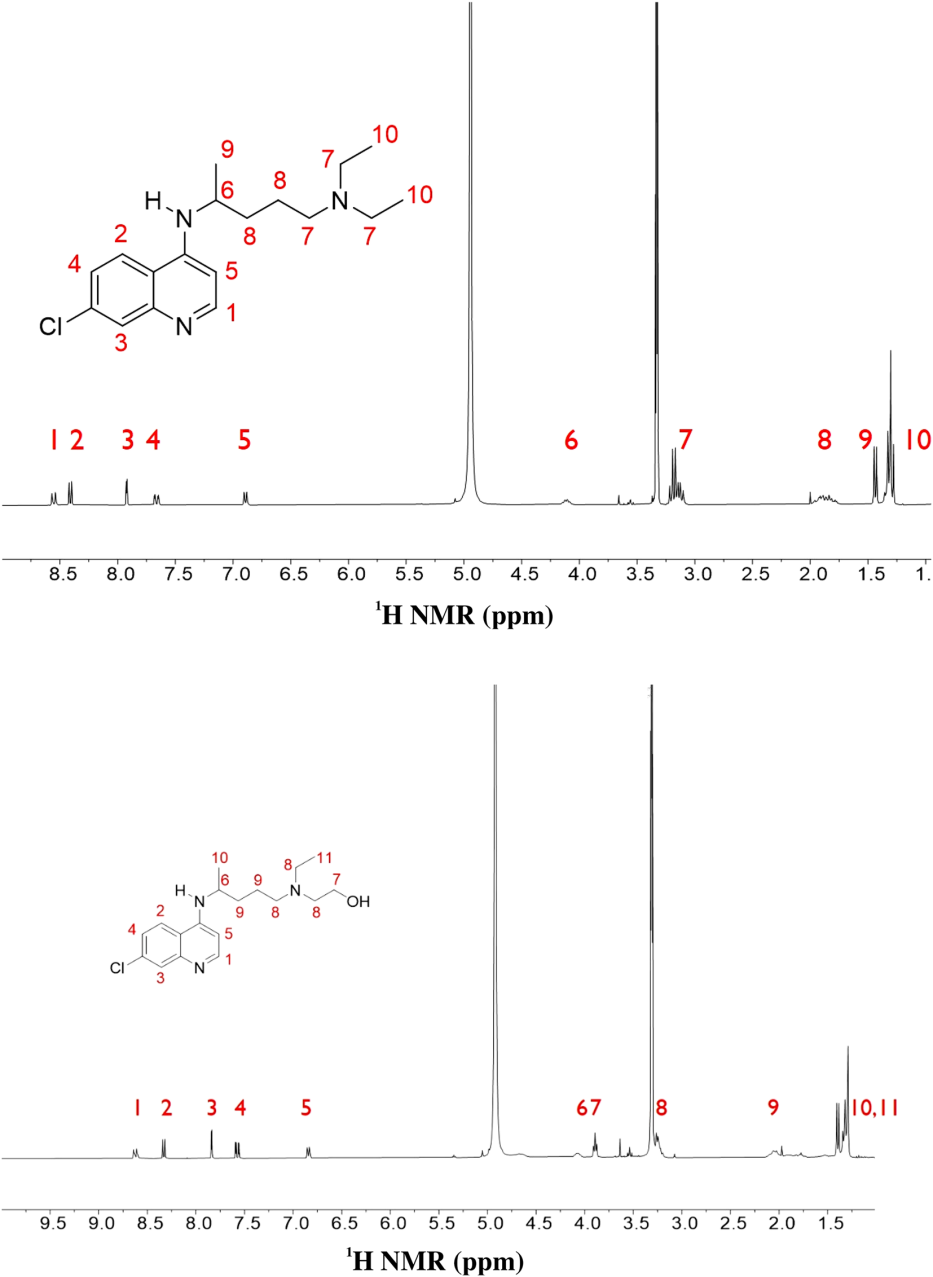
The molecular structure of Chloroquine (upper structure) and Hydroxychloroquine (low structure) molecular structure and those normal ^1^H NMR signals (30° pulse, single scan in 300 MHz NMR).

Chloroquine (hydroxychloroquine) contains a quinoline structure, which may potentially be hyperpolarized using SABRE. However, chloroquine has a long attachment (Fig. 4), which could not be operable as SABRE is dependent on the chemical exchange reaction. It is assumed that the transfer of its binding and kinetics to hyperpolarization with sufficient time from *para*-hydrogen may be challenging. (Fig. 2).

**Figure 5:**
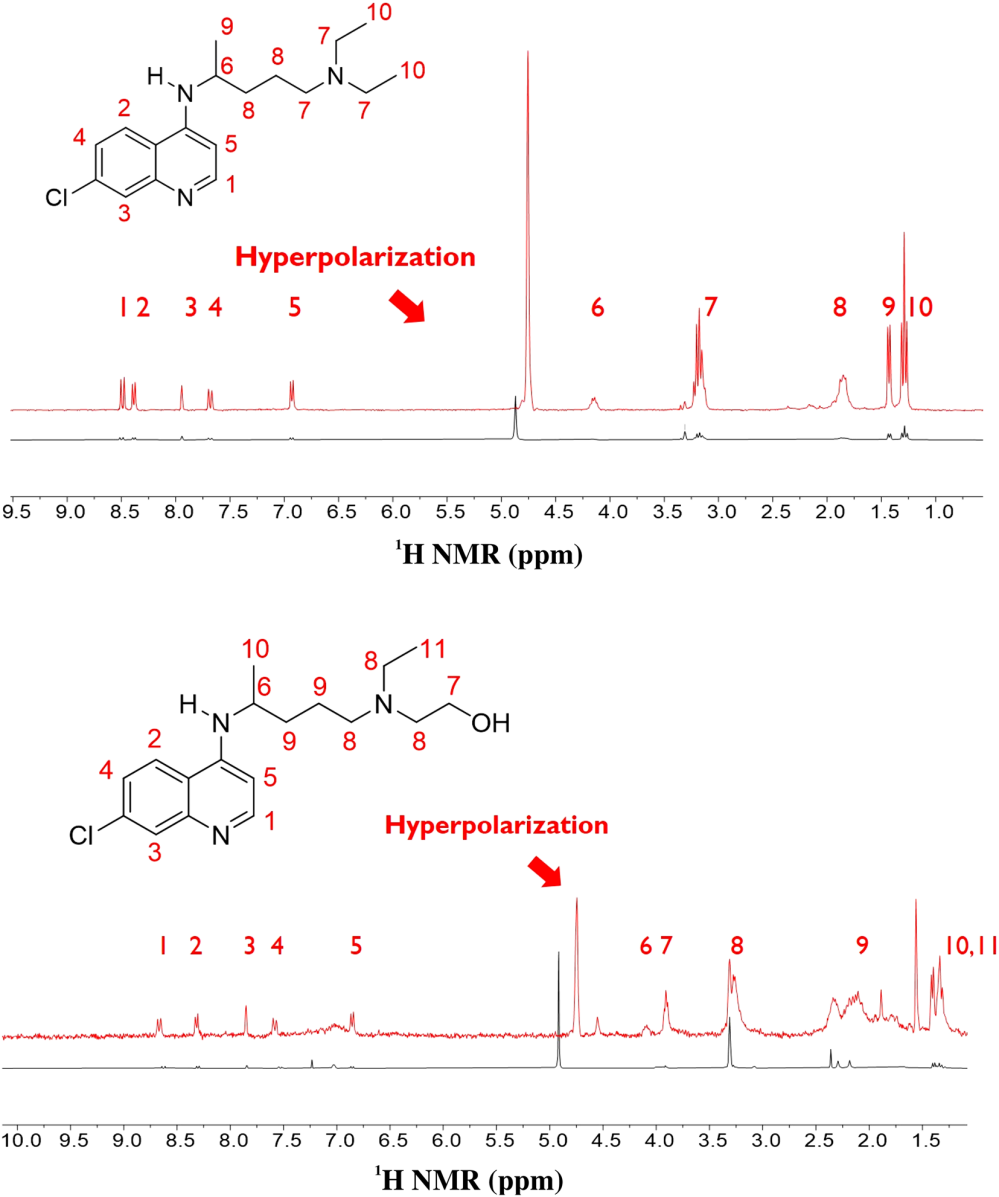
Hyperpolarized signals from chloroquine (upper structure, single scan in 300 MHz ^1^H NMR, 30° pulse of ^1^H after polarization in 90 G magnetic field) and hydroxychloroquine (low structure, single scan in 300 MHz ^1^H NMR, 30° pulse of ^1^H after polarization in 110 G magnetic field) after SABRE.

Interestingly, the polarization transfer from *para*-hydrogen to chloroquine (hydroxychloroquine) was noteworthy as the polarization transfer occurred across a long distance of 11 bonds (from nitrogen to hydrogen number 10). If nitrogen in quinoline is the only group ligating with the Ir catalyst, this is the first observation of such long-range hyperpolarization via SABRE (Fig. 5). It will be worth conducting further studies on the polarization transfer mechanism in SABRE with other molecules, as discussed later in this study.

**Figure 6:**
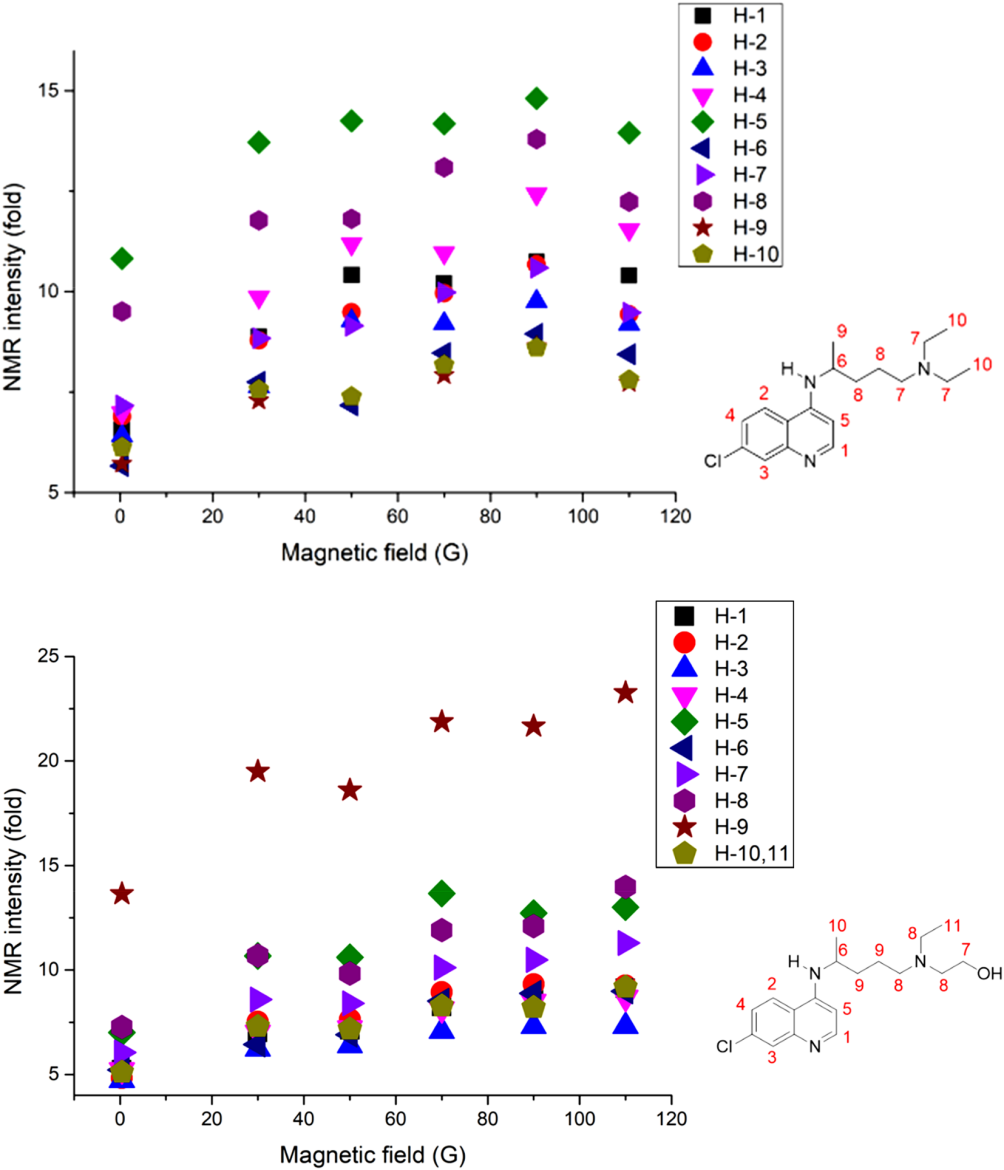
Amplification number of protons from hyperpolarized chloroquine (hydroxychloroquine) structure.

To understand the mechanisms at play, the enhancement of chloroquine (hydroxychloroquine) by hyperpolarization was measured by changing the magnetic field during the polarization transfer period (Fig. 6).

Although the degree of the polarization enhancement was similar, the protons in carbon (H-9 in hydroxychloroquine and H-8 in chloroquine) had the highest and second-highest enhancement, respectively, which differed greatly from the nitrogen in the quinoline moiety. This difference may stem from the direct polarization transfer from the hydride, which bonds to the Ir catalyst through H-9 (hydroxychloroquine) and H-8 (chloroquine) via a dipolar coupling or through the polarization transfer from H-5 to H-9/H-8 by a dipolar coupling or by a J-coupling network^38,39^. The clarification of this mechanism requires further detailed future research. Similarly, the H-5 proton in quinoline on both structures showed high enhancement. These results indicate that the mechanism of polarization transfer is dependent on even a small change in structure and solubility.

It is assumed that the relatively small polarization enhancement can be increased by using higher *para*-hydrogen concentrations, higher partial pressures, and optimal SABRE catalysts^40^. These modifications might drastically improve the enhancement. As Fig. 6 indicates, the polarization was maximized around 70 G, and exhibited a similar trend as that revealed by previous reports on polarization transfer mechanisms^35^.

### Ritonavir/Lopinavir SABRE

**Figure 7:**
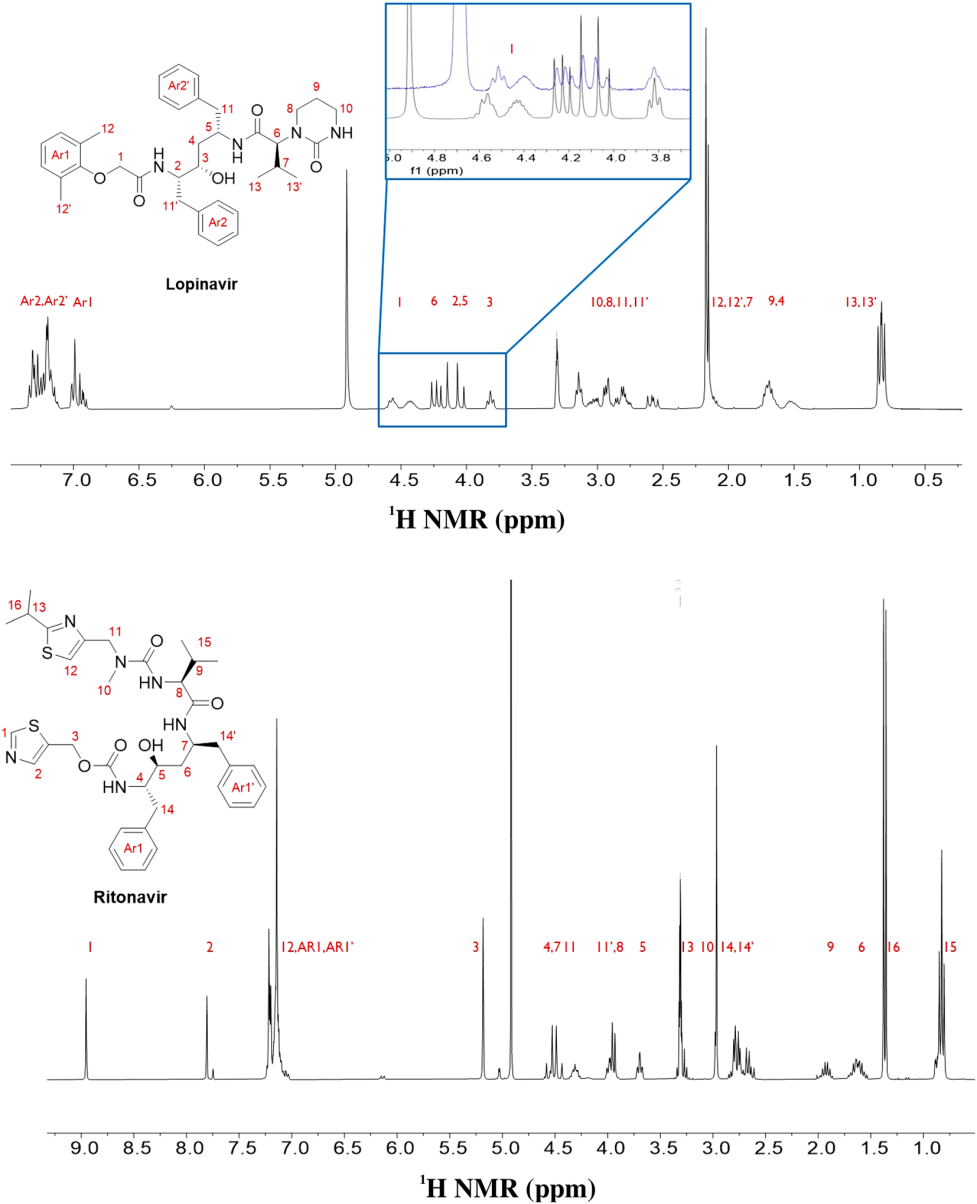
Lopinavir (upper structure) and ritonavir (low structure) molecular structures and their respective normal ^1^H NMR signals (30° pulse, single scan in 300 MHz NMR). The lopinavir proton of **1** shifted after binding with the Ir catalyst (blue spectrum).

Ritonavir/Lopinavir does not contain any well-known functional groups in the structure, which can be harnessed to efficiently undergo polarization transfer from *para*-hydrogen. Furthermore, Lopinavir does not contain sp^2^ nitrogen in the structure, which has been widely used for polarization transfer. However, both structures contain a carbonyl group, which can transfer polarization from *para*-hydrogen to the carbonyl group with the ester group/amide group in the neighborhood. A recent study demonstrated that the carbonyl group can bind to the Ir catalyst, and polarization can be transferred to pyruvate^41^. Moreover, we demonstrated that the Ir catalyst was binding with carbonyl and phenyl ether by detecting the chemical shift of the lopinavir protons of **1** (Fig. 7) after the formation of the complex.

This is the first case in which a binding trend was identified among many functional groups in the structure and, interestingly, polarization was transferred to nearly all protons of lopinavir through this binding site. This behavior should be explored further to understand the polarization transfer mechanism because other functional groups could also participate in binding with the Ir catalyst via a fast exchange, which would mitigate the chemical shift. However, it is noteworthy that the important functional group of lopinavir for polarization transfer could be identified from the chemical shift difference.

**Figure 8:**
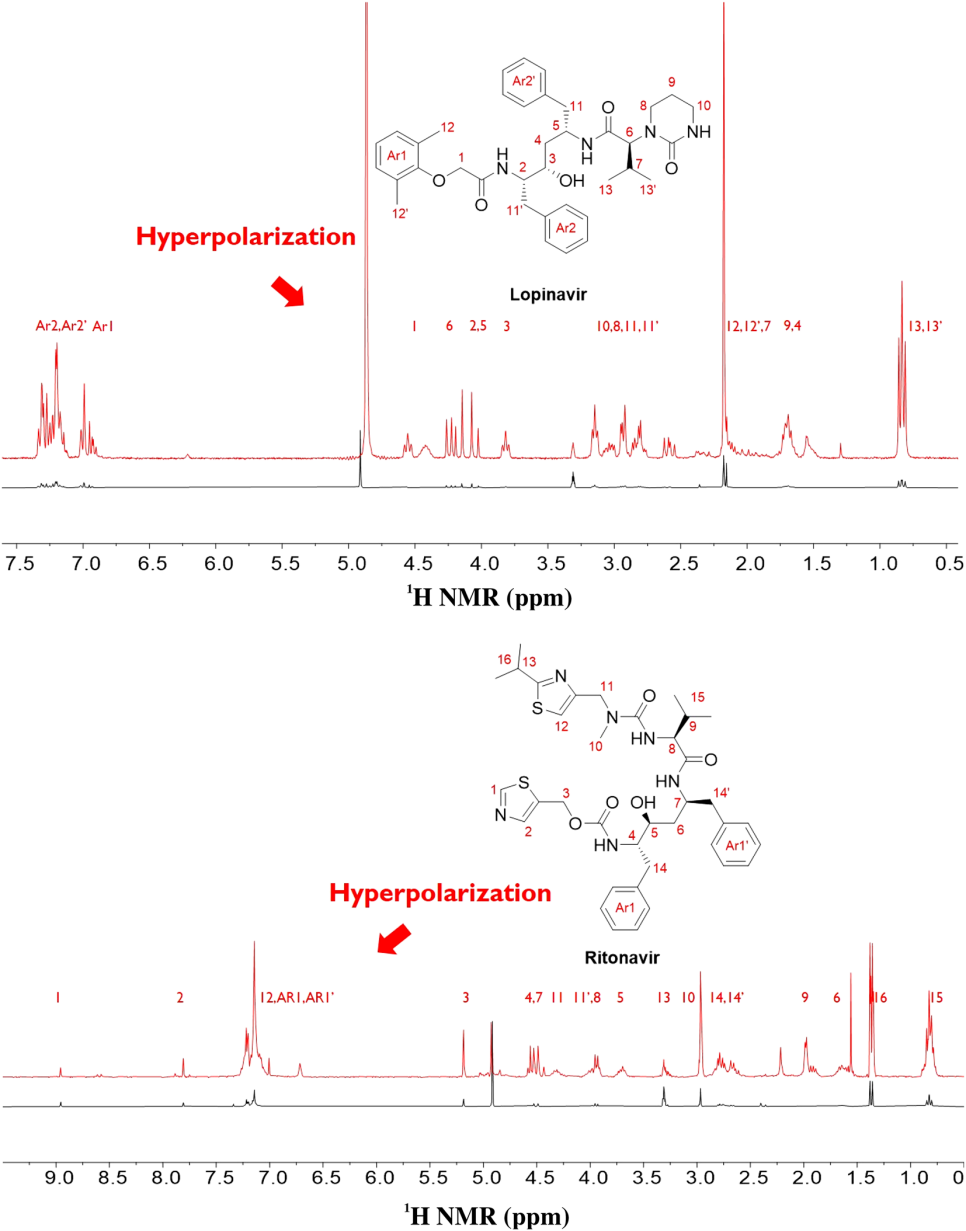
Hyperpolarized signal from lopinavir (upper spectrum) and ritonavir (low spectrum) after SABRE in the presence of a 70 G magnetic field. (single scan in 300 MHz ^1^H NMR, 30° pulse of ^1^H after polarization).

The polarization transfer from *para*-hydrogen to ritonavir/lopinavir is noteworthy as the polarization transferred across a long distance of more than 12 bonds in lopinavir. This is also the first case in which extremely long-range hyperpolarization via SABRE was observed after binding with the carbonyl group (Fig. 8).

**Figure 9:**
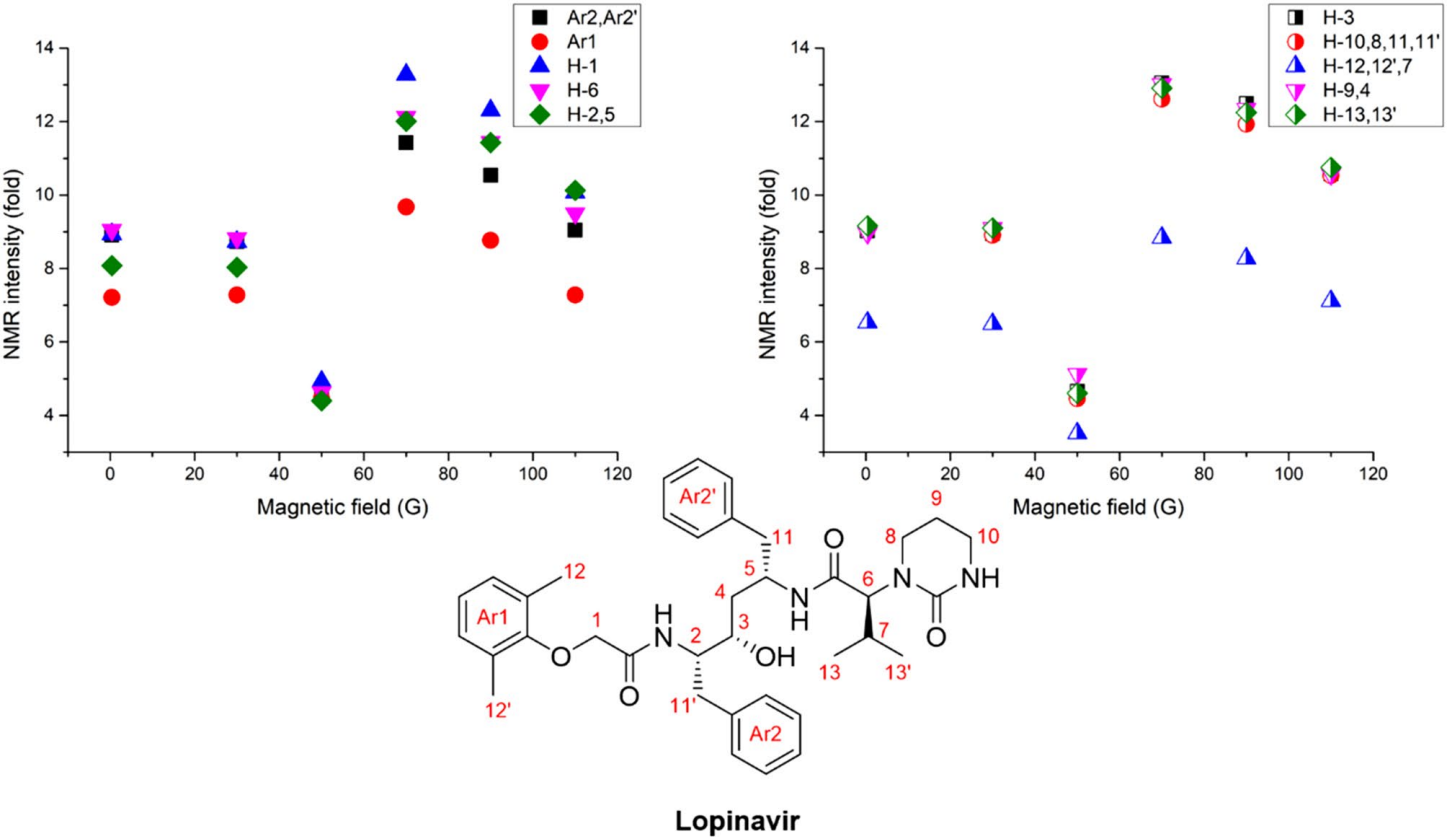
Amplification number of protons from hyperpolarized lopinavir structure.

The enhancement of the proton NMR signal on lopinavir by hyperpolarization was measured by changing the magnetic field in the polarization transfer period to understand the SABRE mechanism (Fig. 9).

This enhancement was also relatively small; however, it was expected to increase when higher *para*-hydrogen concentrations and partial pressures were used. As Fig. 9 shows, polarization was maximized around 70 G, which exhibits a similar trend to previous reports on polarization transfer mechanisms, including chloroquine. Interestingly, H-8 and H-10 exhibited the highest polarization enhancement during SABRE. This may be attributed to the dipolar coupling, the polarization transfer through space, and the SABRE-RELAY mechanism. Understanding the exact mechanism will require further detailed studies in the future^24^.

**Figure 10:**
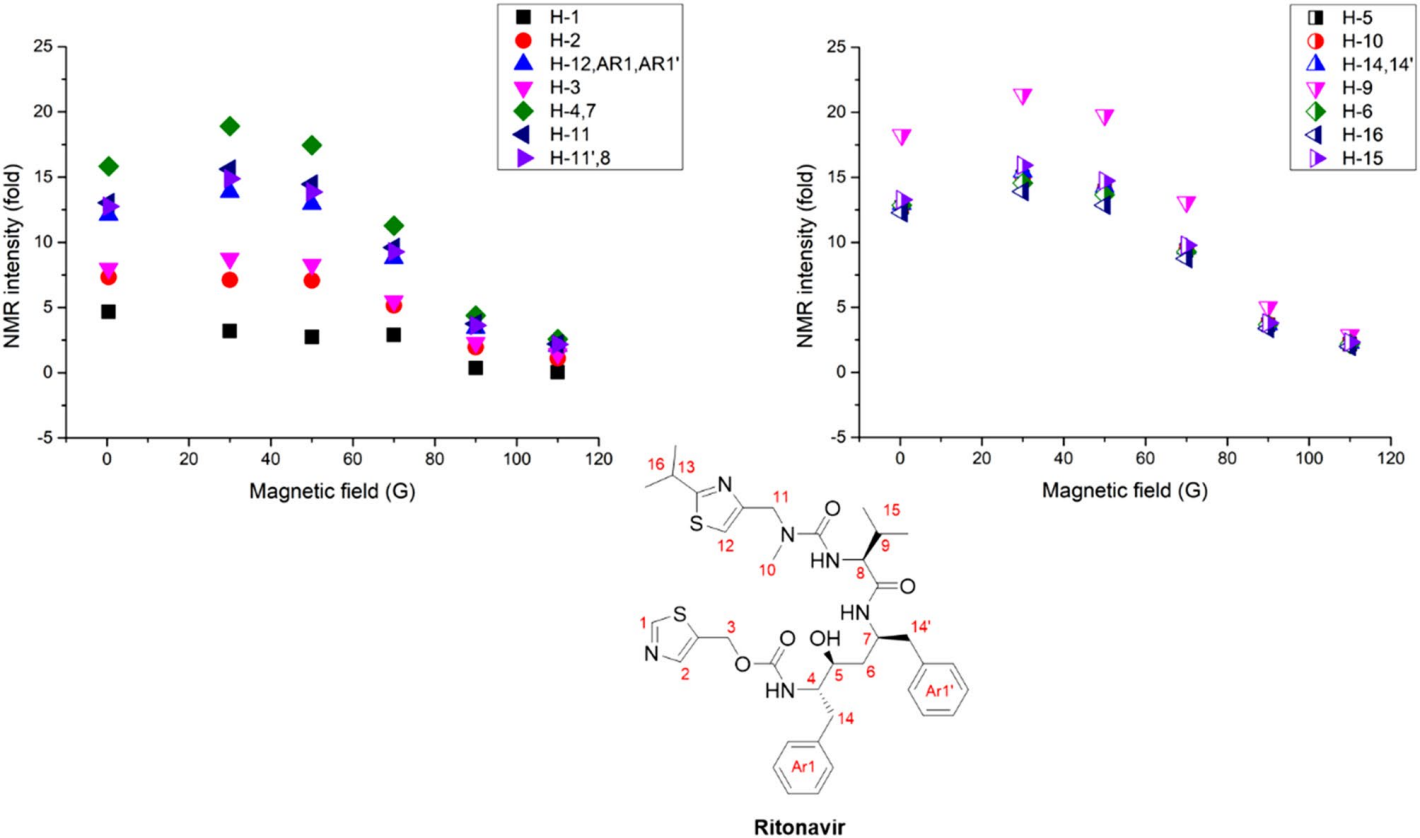
Amplification number of protons from hyperpolarized ritonavir structure.

In terms of the results associated with ritonavir SABRE, 30–50 G was the optimum external magnetic field to match the polarization transfer (Fig. 10). This is a low magnetic field compared to the results of the previous antiviral drug SABRE, which indicates that the optimum magnetic field for polarization transfer is dependent on the binding site with the Ir catalyst and the structure, which can induce different scalar coupling constants. As such, we need to optimize the magnetic field to yield the maximum polarization enhancement in different materials, including the target materials, the solvent, and the catalysts. It was difficult to identify the binding site of ritonavir with the Ir catalyst, as there were several possible binding sites to consider. We observed a small chemical shift in almost all protons of ritonavir after mixing with the Ir catalyst. This implies that there may be more than one binding site for ritonavir with the Ir catalyst. This finding is supported by that there are various possible binding sites, such as two thiazole moieties and three carbonyl sites. Despite the presence of several binding sites in ritonavir, note that hyperpolarization occurred on all protons in the structure, and its enhancement of polarization was almost identical in the higher field. This offers a good explanation for the presence of several binding sites with the Ir catalyst. Ritonavir’s polarization characteristics in the whole structure were only attributable to long-range polarization transfer, the main polarization transfer mechanism for SABRE.

These long-range polarization transfers imply ritonavir’s potential use with hyperpolarized signals in NMR/MRI. This may provide a better understanding of the molecular dynamics of targeted proteins and pharmacokinetics. Importantly, it is anticipated that it may be used for tracking the hyperpolarized signal through MRI. Examples of this include isotope labeling (such as carbon to ^13^C isotope and nitrogen into ^15^ N), in the structures of the studied drugs, which may have a long T1 time. This is not only due to the lower gyromagnetic ratio but also because of its smaller relaxation effect from the Ir catalyst owing to its relatively unstable complex structure.

## Methods

Favipiravir, hydroxychloroquine sulfate, and ritonavir were purchased from Shanghai Alkynechem Co., Ltd. (Shanghai, China) and were used without further purification. Chloroquine diphosphate salt (98.5%) and lopinavir (98%) were acquired from Sigma-Aldrich and were used as received. Methanol-*d*_*4*_(CD_3_OD, 99.8 atom % D, Eurisotop) was also used in the form it was obtained. ^1^H NMR spectra used for the characterization of favipiravir, chloroquine, hydroxychloroquine, lopinavir, and ritonavir were acquired on a Bruker Avance Ш NMR spectrometer operating at a ^1^H resonance frequency of 300 MHz, and were referenced to the residual CH_3_ peak of CD_3_OD (δ = 3.31). Hyperpolarization studies were conducted in the same manner. A home-built instrument was designed as a *para*-hydrogen generator, in which hydrogen gas (Hanmi gas, > 99.9%, a mixture of the spin isomers *ortho*-hydrogen and *para*-hydrogen) was allowed to pass through a heat exchanger filled with a FeO(OH) catalyst (Sigma Aldrich)^42^. This structure was filled with liquid nitrogen in a Dewar flask and produced ca. 50% *para*-hydrogen. In each experiment, *para*-hydrogen continuously flowed into the drug sample at a rate of 6 mL/min at 23 °C and 1 atm. To obtain various magnetic field data, the following system was established and developed: the power supply was GPS-1850D (Bench Power Supply, Linear DC). A shielded coil wound with copper-coated wire and a shielded coil outside the first consisted of a 200 mm diameter and 190 mm height. The magnetic field through the shielded coil was regulated by setting the current, which was in the range of 0–5 A. The magnetic field generated by the controlled current was measured using a Lakeshore Gaussmeter.

Favipiravir (5 mg, 3.1 × 10^−2^ mmol) and the pre-catalyst ([Ir(IMes)(COD)Cl], 2 mg, 3.1 × 10^−3^ mmol) were dissolved in CD_3_OD (900 μL)^43^. Chloroquine and hydroxychloroquine samples for hyperpolarization were prepared by mixing a solution of the substrate (3.9 × 10^−3^ mmol) and [Ir(IMes)(COD)Cl] (2 mg, 3.1 × 10^−3^ mmol) in CD_3_OD (900 μL). Lopinavir and ritonavir (1.6 × 10^−2^ mmol) were added to the CD_3_OD (900 μL) solution of the pre-catalyst ([Ir(IMes)(COD)Cl], 2 mg, 3.1 × 10^−3^ mmol). The mixture of each drug and the pre-catalyst was bubbled by *para*-hydrogen for 20 min in the NMR tube under the earth’s magnetic field for activation. Afterwards, in order to induce hyperpolarization on each sample in each magnetic field for SABRE, the sample was swiftly (less than 5 s) moved directly into a 300 MHz NMR spectrometer and each hyperpolarized proton signal was obtained. All the NMR spectra were then acquired with 1 scan (during 6 s) in a varying magnetic field after polarization of the substrate for 1 min by 50% *para*-hydrogen bubbled at ~ 23 °C under 1 atm. (Earth’s magnetic field, 30 G, 50 G, 70 G, 90 G, and 110 G, respectively). The diverse experiments of the substrate were conducted in the same manner as mentioned above. Subsequently, for calculation of the ^1^H signal enhancement factor (fold), the following equation was used^44,45^:

$$Enhancement\;factor\left( {fold} \right) \, = \, signal\left( A \right){/}signal\left( {non-A} \right)$$

Signal(A) = signal of the amplified sample through hyperpolarization, signal(non-A) = signal of the non-amplified sample (normal ^1^H NMR signal, i.e., Figs. 1, 4, 7). Spectra were acquired on the same sample using duplicate conditions, such as acquisition parameters and receiver gain except *para-*hydrogen usage. The raw integrals of the hyperpolarized and non-hyperpolarized spectra were used to calculate the amplification. To determine the exact integral of the signal through the same chemical shift region, the solvent peak was compared.

## Conclusions

Antiviral drugs, such as chloroquine, hydroxychloroquine, ritonavir, lopinavir, and favipiravir have been investigated as drug candidates in response to the COVID-19 pandemic situation. Spin hyperpolarization may open new opportunities in the diagnosis and biomedical research of COVID-19 via MRI and pharmacodynamics, metabolomics, and binding dynamics with proteins. This can be achieved by using enhanced signal intensity in NMR/MRI. Furthermore, even in case these drug candidates, which are under clinical investigation, may not be useful for the COVID-19, this SABRE-based hyperpolarization study based on high molecular weight structures has not been previously conducted due to the special polarization transfer mechanism. In this study, high molecular weight chloroquine, hydroxychloroquine, ritonavir, lopinavir, and favipiravir were successfully tested for SABRE-based hyperpolarization, and polarizations over long distances were detected, which may result in that the technique will be used for other materials. Understanding the exact polarization transfer mechanism is important for future studies. Hence, other polarization transfer mechanisms, such as those involving binding to other functional groups that are closer to the hyperpolarized spins, SABRE-RELAY should also be investigated through the solvent or relaxation through dipolar effects. Each polarization transfer-maximized external magnetic field was slightly different, which clearly implies that each structure has different optimal polarization transfer matching conditions. Therefore, matching the magnetic field should be controlled to obtain efficient polarization enhancement in each structure. Importantly, this method can be used to monitor the distribution and activity of a drug in vivo by MRI. It may be harnessed to further investigate the molecular interaction of additional drug candidates with key proteins and unveil unknown activity on COVID-19, including pharmacokinetics.

The polarization of the proton may be transferred to other isotope nuclei using pulse sequence, field cycling, and the matching of the spin energy. In the future, this will address the ultimate objective of obtaining hyperpolarized antiviral drugs to study their effect on COVID-19 with a sufficiently long time of T1.

This study was unable to shed significant light on the real applications of the treatment of COVID-19. However, many related studies have reported applications in drug discovery, detecting tumors in vivo, as well as polarization transfer into many other isotopes along with pulse sequence development. Future work on isotope labeling and further polarization transfer on long T1 time nuclei, including clinical perspectives, may open new opportunities to overcome this global pandemic.

## Data Availability

The datasets generated during and/or analyzed during the current study are available from the corresponding author (K. J.) on reasonable request. All methods were performed in accordance with the relevant guidelines and regulations.

## References

[1] Huang C (2020). Clinical features of patients infected with 2019 novel coronavirus in Wuhan, China. Lancet.

[2] Wang M (2020). Remdesivir and chloroquine effectively inhibit the recently emerged novel coronavirus (2019-nCoV) in vitro. Cell Res..

[3] Spinelli FR, Ceccarelli F, Di Franco M, Conti F (2020). Response to ‘is there a future for hydroxychloroquine/chloroquine in prevention of SARS-CoV-2 infection (COVID-19)? by Moiseev et al. Ann. Rheum. Dis..

[4] Guastalegname M, Vallone A (2020). Could chloroquine /hydroxychloroquine be harmful in Coronavirus Disease 2019 (COVID-19) treatment?. Clin. Infect. Dis..

[5] Yan Y (2013). Anti-malaria drug chloroquine is highly effective in treating avian influenza A H5N1 virus infection in an animal model. Cell Res..

[6] Torjesen I (2020). Covid-19: Hydroxychloroquine does not benefit hospitalised patients, UK trial finds. BMJ.

[7] Vincent MJ (2005). Chloroquine is a potent inhibitor of SARS coronavirus infection and spread. Virol. J..

[8] Savarino A, Shytaj IL (2015). Chloroquine and beyond: Exploring anti-rheumatic drugs to reduce immune hyperactivation in HIV/AIDS. Retrovirology.

[9] Mackenzie AH (1983). Dose refinements in long-term therapy of rheumatoid arthritis with antimalarials. Am. J. Med..

[10] Zheng J (2020). SARS-CoV-2: an emerging coronavirus that causes a global threat. Int. J. Biol. Sci..

[11] Yao T, Qian J, Zhu W, Wang Y, Wang G (2020). A systematic review of lopinavir therapy for SARS coronavirus and MERS coronavirus—A possible reference for coronavirus disease-19 treatment option. J. Med. Virol..

[12] Wang Z, Chen X, Lu Y, Chen F, Zhang W (2020). Clinical characteristics and therapeutic procedure for four cases with 2019 novel coronavirus pneumonia receiving combined Chinese and Western medicine treatment. Biosci. Trends.

[13] Cao B (2020). A trial of lopinavir–ritonavir in adults hospitalized with severe Covid-19. N. Engl. J. Med..

[14] Lim J (2020). Case of the index patient who caused tertiary transmission of coronavirus disease 2019 in Korea: The application of lopinavir/ritonavir for the treatment of COVID-19 pneumonia monitored by quantitative RT-PCR. J. Korean Med. Sci..

[15] Kim JY (2020). The first case of 2019 novel coronavirus pneumonia imported into Korea from Wuhan, China: Implication for infection prevention and control measures. J. Korean Med. Sci..

[16] Goldhill DH (2018). The mechanism of resistance to favipiravir in influenza. Proc. Natl. Acad. Sci. USA.

[17] Hayden FG, Shindo N (2019). Influenza virus polymerase inhibitors in clinical development. Curr. Opin. Infect. Dis..

[18] Chen C (2020). Favipiravir versus arbidol for COVID-19: A randomized clinical trial. medRxiv.

[19] Tanner CPN (2019). Selective hyperpolarization of heteronuclear singlet states via pulsed microtesla SABRE. J. Chem. Phys..

[20] Barskiy DA (2016). Over 20% 15N Hyperpolarization in under one minute for metronidazole, an antibiotic and hypoxia probe. J. Am. Chem. Soc..

[21] Glachet T (2019). Iodonitrene in Action: Direct transformation of amino acids into terminal diazirines and 15N2-diazirines and their application as hyperpolarized markers. J. Am. Chem. Soc..

[22] Theis T (2015). Microtesla SABRE enables 10% nitrogen-15 nuclear s. J. Am. Chem. Soc..

[23] Truong ML (2015). 15N Hyperpolarization by reversible exchange using SABRE-SHEATH. J. Phys. Chem. C.

[24] Iali W, Rayner PJ, Duckett SB (2018). Using parahydrogen to hyperpolarize amines, amides, carboxylic acids, alcohols, phosphates, and carbonates. Sci. Adv..

[25] Ariyasingha NM (2019). Quasi-resonance fluorine-19 signal amplification by reversible exchange. J. Phys. Chem. Lett..

[26] Olaru AM (2016). Using signal amplification by reversible exchange (SABRE) to hyperpolarise 119Sn and 29Si NMR nuclei. Chem. Commun..

[27] Rayner PJ, Richardson PM, Duckett SB (2020). The detection and reactivity of silanols and silanes using hyperpolarized 29 Si nuclear magnetic resonance. Angew. Chemie.

[28] Iali W (2019). Hyperpolarising pyruvate through signal amplification by reversible exchange (SABRE). Angew. Chemie Int. Ed..

[30] Richards JE (2018). Using hyperpolarised NMR and DFT to rationalise the unexpected hydrogenation of quinazoline to 3,4-dihydroquinazoline. Chem. Commun..

[30] Ratajczyk T (2015). NMR signal enhancement by effective SABRE labeling of oligopeptides. Chem. A Eur. J..

[31] Svyatova A (2019). 15N MRI of SLIC-SABRE Hyperpolarized 15N-labelled pyridine and nicotinamide. Chem. A Eur. J..

[32] Robertson TBR (2019). Hyperpolarization of pyridyl fentalogues by signal amplification by reversible exchange (SABRE). ChemistryOpen.

[33] Linnik IV, Rayner PJ, Stow RA, Duckett SB, Cheetham GMT (2019). Pharmacokinetics of the SABRE agent 4,6-d 2 -nicotinamide and also nicotinamide in rats following oral and intravenous administration. Eur. J. Pharm. Sci..

[34] Eshuis N (2016). Determination of long-range scalar 1H–1H coupling constants responsible for polarization transfer in SABRE. J. Magn. Reson..

[35] Atkinson KD (2009). Spontaneous transfer of Parahydrogen derived spin order to pyridine at low magnetic field. J. Am. Chem. Soc..

[36] Adams RW (2009). Reversible interactions with para-hydrogen enhance NMR sensitivity by polarization transfer. Science (80-. ).

[37] Daniele V, Legrand FX, Berthault P, Dumez JN, Huber G (2015). Single-Scan Multidimensional NMR Analysis of mixtures at sub-millimolar concentrations by using SABRE hyperpolarization. ChemPhysChem.

[38] Barskiy DA (2014). The feasibility of formation and kinetics of NMR signal amplification by reversible exchange (SABRE) at high magnetic field (9.4 T). J. Am. Chem. Soc..

[39] Shchepin RV (2017). Spin relays enable efficient long-range heteronuclear signal amplification by reversible exchange. J. Phys. Chem. C.

[40] Colell J (2020). Rational ligand choice extends the SABRE substrate scope. Chem. Commun..

[41] Tickner BJ (2020). Optimisation of pyruvate hyperpolarisation using SABRE by tuning the active magnetisation transfer catalyst. Catal. Sci. Technol..

[42] Jeong K, Min S, Chae H, Namgoong SK (2019). Monitoring of hydrogenation by benchtop NMR with parahydrogen-induced polarization. Magn. Reson. Chem..

[43] Lee SJ (2019). SQUID-based ultralow-field MRI of a hyperpolarized material using signal amplification by reversible exchange. Sci. Rep..

[44] Jeong HJ, Min S, Jeong K (2020). Analysis of 1-aminoisoquinoline using the signal amplification by reversible exchange hyperpolarization technique. Analyst.

[45] Chae H (2020). Organic reaction monitoring of a glycine derivative using signal amplification by reversible exchange-hyperpolarized benchtop nuclear magnetic resonance spectroscopy. Anal. Chem..

